# Supplementary material for: Chronic depletion of vertebrate lipids in Aedes aegypti cells dysregulates lipid metabolism and inhibits innate immunity without altering dengue infectivity
Source: PLoS Negl Trop Dis. 2022 Oct 24;16(10):e0010890. doi: 10.1371/journal.pntd.0010890 (PMC9632908; doi:10.1371/journal.pntd.0010890)
Supplement: S6 Table — (DOCX) [file pntd.0010890.s006.docx]

S6 Table. Primers for RT-qPCR targets

| Name | Identifier | Forward Primer | Reverse Primer |
| --- | --- | --- | --- |
| TOLL5A | AAEL007619 | 5’ GGAGCACGGACTTCAGTTCA ‘3 | 5’ GTAGCATCGAGGAGGTTCGG ’3 |
| Yellow-d2 | AAEL004863 | 5’ TTCGACTTGCGAACCGATCA ‘3 | 5’ GAATGCTGACAAGCAGCGAG ‘3 |
| SRPN3 | AAEL005665 | 5’ AAGGACTTTGGGTCAACGTG ‘3 | 5’ ATGCGGTTCTAAATGGTTGG ‘3 |
| SRPN27A | AAEL014078 | 5’ GTGATCTGAATGCAACCCGC ‘3 | 5’ TGGGAAGACAGTGCCGAATC ‘3 |
