# Supplementary material for: Chronic depletion of vertebrate lipids in Aedes aegypti cells dysregulates lipid metabolism and inhibits innate immunity without altering dengue infectivity
Source: PLoS Negl Trop Dis. 2022 Oct 24;16(10):e0010890. doi: 10.1371/journal.pntd.0010890 (PMC9632908; doi:10.1371/journal.pntd.0010890)
Supplement: Attachment — Submitted filename: PNTD-D-22-00671_R1_reviewer.pdf [file pntd.0010890.s009.pdf]

PLOS Neglected Tropical Diseases

Chronic depletion of vertebrate lipids in Aedes aegypti cells dysregulates lipid metabolism and inhibits innate immunity without altering dengue infectivity

--Manuscript Draft--

**Manuscript Number:**	PNTD-D-22-00671R1
**Full Title:**	Chronic depletion of vertebrate lipids in Aedes aegypti cells dysregulates lipid metabolism and inhibits innate immunity without altering dengue infectivity
**Short Title:**	Chronic depletion of lipids in Aedes aegypti
**Article Type:**	Research Article
**Keywords:**	Aedes aegypti; Blood; cholesterol; Fatty acids; Triglyceride; Lipid droplet; innate immunity; Dengue
**Abstract:**	Aedes aegypti is the primary vector of dengue virus (DENV) and other arboviruses. Previous literature suggests that vertebrate and invertebrate lipids and the nutritional status of mosquitoes modify virus infection. Here, we developed a vertebrate lipid-depleted Ae. aegypti cell line to investigate if chronic depletion of vertebrate lipids normally present in a blood meal and insect cell culture medium would impact cell growth and virus infection. Chronic depletion of vertebrate lipids reduced cell size and proliferation, although cells retained equivalent total intracellular lipids per cell by reducing lipolysis and modifying gene expression related to sugar and lipid metabolism. Downregulation of innate immunity genes was also observed. We hypothesized that chronic depletion of vertebrate lipids would impact virus infection; however, the same amount of DENV was produced per cell. This study reveals how Ae. aegypti cells adapt in the absence of vertebrate lipids, and how DENV can replicate equally well in cells that contain predominately vertebrate or invertebrate lipids.
**Additional Information:**	
**Question**	**Response**
**Financial Disclosure**<br><br>Enter a financial disclosure statement that describes the sources of funding for the work included in this submission. Review the submission guidelines for detailed requirements. View published research articles from *PLOS NTDs* for specific examples.<br><br>This statement is required for submission and **will appear in the published article** if the submission is accepted. Please make sure it is accurate.	This work was supported by bridge funding from Central Michigan University College of Medicine.

Powered by Editorial Manager® and ProduXion Manager® from Aries Systems Corporation

**Unfunded studies**<br><br>Enter: *The author(s) received no specific funding for this work.*<br><br>**Funded studies**<br><br>Enter a statement with the following details:<br>• Initials of the authors who received each award<br>• Grant numbers awarded to each author<br>• The full name of each funder<br>• URL of each funder website<br>• Did the sponsors or funders play any role in the study design, data collection and analysis, decision to publish, or preparation of the manuscript?<br>• **NO** - Include this sentence at the end of your statement: *The funders had no role in study design, data collection and analysis, decision to publish, or preparation of the manuscript.*<br>• **YES** - Specify the role(s) played.<br><br>* typeset	
**Competing Interests**<br><br>Use the instructions below to enter a competing interest statement for this submission. On behalf of all authors, disclose any competing interests that could be perceived to bias this work—acknowledging all financial support and any other relevant financial or non-financial competing interests.<br><br>This statement **will appear in the published article** if the submission is accepted. Please make sure it is accurate. View published research articles from *PLOS NTDs* for specific examples.	The authors have declared that no competing interests exist.

Powered by Editorial Manager® and ProduXion Manager® from Aries Systems Corporation

**NO authors have competing interests**<br><br>Enter: *The authors have declared that no competing interests exist.*<br><br>**Authors with competing interests**<br><br>Enter competing interest details beginning with this statement:<br><br>*I have read the journal's policy and the authors of this manuscript have the following competing interests: [insert competing interests here]*<br><br>* typeset<br><br>This statement is required for submission and **will appear in the published article** if the submission is accepted. Please make sure it is accurate and that any funding sources listed in your Funding Information later in the submission form are also declared in your Financial Disclosure statement.	
**Data Availability**<br><br>Authors are required to make all data underlying the findings described fully available, without restriction, and from the time of publication. PLOS allows rare exceptions to address legal and ethical concerns. See the PLOS Data Policy and FAQ for detailed information.	Yes - all data are fully available without restriction

Powered by Editorial Manager® and ProduXion Manager® from Aries Systems Corporation

A Data Availability Statement describing where the data can be found is required at submission. Your answers to this question constitute the Data Availability Statement and **will be published in the article**, if accepted.<br><br>**Important:** Stating 'data available on request from the author' is not sufficient. If your data are only available upon request, select 'No' for the first question and explain your exceptional situation in the text box.<br><br>Do the authors confirm that all data underlying the findings described in their manuscript are fully available without restriction?	
**Describe where the data may be found in full sentences. If you are copying our sample text, replace any instances of XXX with the appropriate details.**<br><br>• If the data are **held or will be held in a public repository**, include URLs, accession numbers or DOIs. If this information will only be available after acceptance, indicate this by ticking the box below. For example: *All XXX files are available from the XXX database (accession number(s) XXX, XXX.).*<br>• If the data are all contained **within the manuscript and/or Supporting Information files**, enter the following: *All relevant data are within the manuscript and its Supporting Information files.*<br>• If neither of these applies but you are able to provide **details of access elsewhere**, with or without limitations, please do so. For example:<br><br>*Data cannot be shared publicly because*	Supplemental data.

Powered by Editorial Manager® and ProduXion Manager® from Aries Systems Corporation

of [XXX]. Data are available from the XXX Institutional Data Access / Ethics Committee (contact via XXX) for researchers who meet the criteria for access to confidential data.<br><br>*The data underlying the results presented in the study are available from (include the name of the third party and contact information or URL).*<br>• This text is appropriate if the data are owned by a third party and authors do not have permission to share the data.<br><br>* typeset	
Additional data availability information:	
**Government Employee**<br><br>Are you or any of the contributing authors an employee of the United States government?<br><br>Manuscripts authored by one or more US Government employees are not copyrighted, but are licensed under a CC0 Public Domain Dedication, which allows unlimited distribution and reuse of the article for any lawful purpose. This is a legal requirement for US Government employees.<br><br>This will be typeset if the manuscript is accepted for publication.	No - No authors are employees of the U.S. government.
**Financial Disclosure**<br><br>Enter a financial disclosure statement that describes the sources of funding for the work included in this submission and the role the funder(s) played. This includes grants and any commercial funding of the work or authors.<br><br>This statement will be typeset if the	This work was supported by bridge funding from Central Michigan University College of Medicine. The funders had no role in study design, data collection and analysis, decision to publish, or preparation of the manuscript. Michael Conway, Jesse Bakke, and Maya Tree were employed by Central Michigan University. Andrew Marten and Clara Tift were students.

manuscript is accepted for publication.<br><br>Review the submission guidelines and the instructions link below for detailed requirements and guidance.	
**Competing Interests**<br><br>On behalf of all authors, disclose any competing interests that could be perceived to bias this work.<br><br>This statement will be typeset if the manuscript is accepted for publication.<br><br>Review the instructions link below and PLOS NTDs' competing interests policy to determine what information must be disclosed at submission.	None.
**Data Availability**<br><br>Provide a **Data Availability Statement** in the box below. This statement should detail where the data used in this submission can be accessed. This statement will be typeset if the manuscript is accepted for publication.<br><br>Before publication, authors are required to make all data underlying their findings fully available, without restriction. Review our PLOS Data Policy page for detailed information on this policy. Instructions for writing your Data Availability statement can be accessed via the Instructions link below.	Raw RNA-Seq data files are available at http://www.ncbi.nlm.nih.gov/bioproject/882813 (BioSample accessions: SAMN30952236, SAMN30952237)

Powered by Editorial Manager® and ProduXion Manager® from Aries Systems Corporation

Manuscript Click here to access/download;Manuscript;Manuscript-V2.docx

Chronic depletion of vertebrate lipids in *Aedes aegypti* cells dysregulates lipid metabolism and inhibits innate immunity without altering dengue infectivity

Andrew D. Marten[1], Clara T. Tift[1], Maya O. Tree[1], Jesse Bakke[1], and Michael J. Conway[1#]

[1]Foundational Sciences, Central Michigan University
College of Medicine, Mt. Pleasant, MI

# Address correspondence to: Foundational Sciences, Central Michigan University
College of Medicine, Mount Pleasant, MI, 48859
E-mail: michael.conway@cmich.edu. Phone: (989) 774-3930, Fax: (989) 774-3462

Short title: Chronic depletion of lipids in *Aedes aegypti*

Abstract

Aedes aegypti is the primary vector of dengue virus (DENV) and other arboviruses. Previous literature suggests that vertebrate and invertebrate lipids and the nutritional status of mosquitoes modify virus infection. Here, we developed a vertebrate lipid-depleted *Ae. aegypti* cell line to investigate if chronic depletion of vertebrate lipids normally present in a blood meal and insect cell culture medium would impact cell growth and virus infection. Chronic depletion of vertebrate lipids reduced cell size and proliferation, although cells retained equivalent total intracellular lipids per cell by reducing lipolysis and modifying gene expression related to sugar and lipid metabolism. Downregulation of innate immunity genes was also observed. We hypothesized that chronic depletion of vertebrate lipids would impact virus infection; however, the same amount of DENV was produced per cell. This study reveals how *Ae. aegypti* cells adapt in the absence of vertebrate lipids, and how DENV can replicate equally well in cells that contain predominately vertebrate or invertebrate lipids.

Author Summary

Aedes aegypti is a major threat to public health. *Ae. aegypti* is the primary vector of dengue virus types 1-4 (DENV 1-4), zika virus (ZIKV), chikungunya virus (CHIKV), and yellow fever virus (YFV). *Ae. aegypti* acquires arboviruses from a vertebrate host during blood feeding. Blood feeding introduces vertebrate-specific factors into the mosquito that may be important for both mosquito and virus. This study reveals that *Ae. aegypti* adapts to depletion of vertebrate lipids by inhibiting lipolysis and promoting *de novo* synthesis of invertebrate lipids, and that DENV can replicate equally well without high concentrations of cholesterol and other vertebrate lipid

species. Understanding how disease vectors adapt to nutritional changes will identify novel strategies for vector control and disease mitigation.

Introduction

Vector-borne diseases are a major public health threat, and the risk of infection can increase due to a number of factors including climate change, globalization, pesticide resistance, and deforestation (1–3). *Aedes aegypti* is the primary vector of dengue virus (DENV), zika virus (ZIKV), chikungunya virus (CHIKV), and yellow fever virus (YFV) [1, 2]. There are very few targeted strategies to control *Ae. aegypti*, and there are limited prophylactic and treatment options for infection with the above arboviruses [1]. For example, there are currently no licensed antiviral therapies for DENV, and the currently licensed vaccine is restricted for use in seropositive individuals because of the possible risk of antibody-dependent enhancement (ADE) [3-5]. The severe acute respiratory syndrome coronavirus type 2 (SARS-CoV-2) pandemic has caused additional concern for regions at risk for vector-borne diseases [6]. SARS-CoV-2 may co-infect patients with vector-borne diseases, increasing the number of patients requiring intensive care and mechanical ventilation, especially for those with chronic comorbidities.

Elucidating virus-vector-host interactions that control acquisition and transmission of mosquito-borne viruses is critical in our effort to develop novel prophylactic and antiviral strategies [1, 7]. DENV acquisition in the mosquito begins upon the engorgement of an infected blood meal from a vertebrate host, followed by storage of infected blood in the mosquito midgut. DENV bypasses the peritrophic matrix and forms specific interactions with secreted and

membrane-bound receptors [8-10]. The infectious blood meal is a complex mixture of host proteins, sugars, and lipids. For mosquitoes, the ingested blood meal is an important source of nutrition that supports oogenesis [11]. The lipid fraction contains free fatty acids (FFAs), triacylglycerol, cholesterol, and cell-associated phospholipids, although the contribution of blood meal-derived vertebrate lipids to mosquito physiology and virus infection is largely unknown [8, 11-14].

Previous research has shown that intracellular lipids are important for DENV replication in vertebrate and invertebrate cells and that DENV infection can manipulate the lipidome to promote its replication [15-22]. Importantly, alterations in cholesterol and lipid trafficking either through Wolbachia infection or chemical/genetic manipulation interfered with DENV infection [17, 18, 20, 23]. Our previous studies showed that lipid components in the blood meal inhibit flavivirus acquisition in *Ae. aegypti*. Specifically, human low-density lipoprotein (LDL) entered mosquito cells through clathrin-mediated endocytosis and inhibited flavivirus infection *in vitro* and *in vivo* [8]. Further research revealed that vertebrate lipids in the form of extracellular vesicles (EVs) had no effect on virus cell attachment or entry, but that EVs restricted DENV fusion in the *Ae. aegypti*-derived (Aag2) cell line but not in mammalian cells [13]. Vertebrate lipids appear to interfere with DENV at an early stage in its life cycle. In contrast, DENV reduced protein expression of low-density lipoprotein receptor-related protein 1 (LRP-1) in Aag2 cells leading to increased intracellular cholesterol levels and enhanced virus replication [24]. These studies reveal a complicated relationship with vertebrate lipids. On one hand, vertebrate lipids in a blood meal or cell culture media can inhibit an early stage of infection, yet intracellular lipids may benefit virus replication.

In nature, the composition and frequency of blood meals will vary, which will lead some mosquitoes to experience nutrient deficiency and starvation. These changes in nutritional status may alter mosquito physiology and influence their fitness, susceptibility to insecticides, and virus infection [25-30]. We hypothesized that chronic depletion of vertebrate lipids would influence mosquito cell growth and DENV infection. To test this hypothesis, we generated a vertebrate lipid-depleted Aag2 cell line by feeding cells with lipid-depleted cell culture media. The Aag2 cell line was generated from whole homogenized embryos. Cells within the culture exhibit differing morphologies, and it has been suggested that the varying morphologies indicate the presence of multiple embryonic and differentiated cell types. Aag2 cells are immunocompetent and persistently infected with a number of insect-specific viruses, including cell fusing agent virus (CFAV) and Phasi Charoen-like virus (PCLV) [31-33]. Our study revealed that lipid-depleted (LD) Aag2 (LD-Aag2) cells have reduced cell proliferation and size, although they contain the same amount of intracellular lipids per cell as Aag2 cells that were fed complete cell culture medium. The total amount of intracellular lipids per cell were increased upon addition of excess glucose, suggesting that Aag2 cells can adapt to lipid-depleted conditions by promoting *de novo* lipid biosynthesis. RNA-Seq analysis revealed differential gene expression between the two cell lines and identified clusters of genes involved in sugar and lipid metabolism. Considering the dramatic difference in cell proliferation and size and differential gene expression, which included changes to some innate immunity genes, we hypothesized that LD-Aag2 cells would have a different susceptibility to DENV infection than Aag2 cells that were fed complete cell culture medium. Instead, we found that DENV production was equivalent in both cell types. These results imply that mosquito cells can adapt to lipid-depleted conditions by

promoting *de novo* lipid biosynthesis and that DENV can replicate equally well in mosquito cells that contain vertebrate or invertebrate lipids.

Results

Establishment of a lipid-depleted (LD) *Aedes aegypti* cell line

In order to test the role of cholesterol and other vertebrate lipids in mosquito cell metabolism and infection, we established a lipid-depleted (LD) *Ae. aegypti* cell line by feeding cells with LD cell culture media. LD cell culture media was made with fetal bovine serum (FBS) that had been extracted with fumed silica [24, 34, 35]. Fumed silica removes cholesterol, phosphatidylcholines, and triacyl glycerides from serum with minimal removal of fatty acids and essential proteins such as albumin [34, 35]. We confirmed that fumed silica removed cholesterol from FBS by quantifying cholesterol concentration in complete (C) and LD cell culture media. Cholesterol concentration was significantly lower in LD cell culture media **(Figure 1A**). Additionally, we showed that intracellular cholesterol concentration rapidly declined over the course of one week when cells were treated with LD cell culture media (**Figure 1B)**. Free fatty acid (FFA) concentration was quantified in C and LD cell culture media, and FFAs were reduced in LD cell culture media by approximately 50 percent (**Figure 1C**). Triacylglycerol (TAG) concentration was quantified in C and LD cell culture media, and TAGs were reduced in LD cell culture media by approximately 15 percent (**Figure 1D**). Cell proliferation stalled for two months while feeding with LD cell culture media, although the morphology of cells treated with

C and LD cell culture media were equivalent after this time frame (**Figure 1E**), and LD-Aag2 cells began to proliferate, albeit at a lower rate than C-Aag2 cells (**Figure 1F**). LD-Aag2 cells were expanded approximately three months after initiation of this cell line and passage zero cells were frozen at -80°C. LD-Aag2 cells were rarely grown past passage 6.

Ae. aegypti are sterol auxotrophs and derive this lipid from vertebrate blood and other sources [20, 24, 36]. We expected that depriving cells of vertebrate lipids would reduce intracellular cholesterol concentration. We hypothesized that LD-Aag2 cells would adapt by synthesizing fatty acids to compensate for the loss of sterols and key vertebrate lipids in their nutrition. To test this hypothesis, we quantified the total intracellular lipid content of C and LD-Aag2 cells using a lipophilic and fluorescent Nile Red stain and nuclear Hoechst counter stain [37]. Hoechst counter stain was used to normalize total intracellular lipid signal per cell. Additionally, we fed LD-Aag2 six times (6X) normal glucose concentration to see if LD-Aag2 cells utilized glucose for *de novo* lipid biosynthesis. Representative Nile Red fluorescent images revealed that LD-Aag2 and LD-6X-Aag2 cells contained at least as much total lipid per cell as C-Aag2 cells (**Figure 2A**). We quantified the area of each cell and determined that LD-Aag2 cells were significantly smaller than C-Aag2, although treatment with LD-6X glucose cell culture medium reverted cell size to normal (**Figure 2B**). We then used pixel intensity as a proxy for lipid concentration and determined that C-Aag2 and LD-Aag2 cells had the same amount of total intracellular lipid per cell. Interestingly, addition of LD-6X cell culture media significantly increased the total intracellular lipid per cell (**Figure 2C**). We hypothesized that LD-Aag2 cells maintain similar total intracellular lipids per cell by reducing lipolysis of triglycerides stored in lipid droplets. We tested this hypothesis by performing a glycerol analysis of C-Aag2 cell free

supernatants after treatment with C or LD cell culture media for seven days. Lipolysis converts triglycerides into one glycerol and three fatty acid molecules. Production of glycerol fell sharply at days 5 and 7 post-treatment with LD cell culture media (**Figure 2D**).

Cell proliferation of LD-Aag2 cells was reduced despite cells containing equivalent amounts of total intracellular lipids per cell. We hypothesized that LD-Aag2 cells used glucose and remaining FFAs for *de novo* lipid biosynthesis rather than for ATP generation. To test this hypothesis, we quantified intracellular ATP concentration in cells three days post-treatment with C and LD cell culture media. There was no difference in intracellular ATP concentration per cell (**Figure 3**).

RNA-Seq analysis summary

LD-Aag2 cells were depleted of intracellular cholesterol, yet cells treated with C and LD cell culture media contained the same concentrations of total intracellular lipid per cell. Similarly, there was no difference in the total concentration of intracellular ATP. However, we did find that cell proliferation in LD-Aag2 cells was reduced. We hypothesized that Aag2 adapt to lipid-depleted conditions by changing expression of metabolism genes, which would reduce mitotic rate to match the reduction in catabolic substrate and promote *de novo* lipid biosynthesis.

We tested this hypothesis by performing next generation RNA sequencing (RNA-Seq) analysis on C and LD-Aag2 cells and investigated the statistically significant differentially expressed genes (DEGs). Volcano plot analysis revealed 776 genes that were upregulated and

689 genes that were downregulated in LD-Aag2 cells (**Figure 4**). RNA-Seq data can be seen in **Supplementary Tables 1-3**.

The g:Cost Functional Profiling tool at g:Profiler was used to quantify the number of genes that belong to specific categories that were either upregulated or downregulated in LD-Aag2 cells. Only unique identifiers and not “novel” genes identified by RNA-Seq were used for analysis. The gene ontology (GO) molecular function, biological process, and cellular compartment categories with the highest number of intersecting upregulated LD-Aag2 genes were hydrolase activity, proteolysis, and membrane, respectively (**Figure 5A-C**). The molecular function, biological process, and cellular compartment categories with the highest number of intersecting downregulated LD-Aag2 genes were transmembrane transporter activity, localization, and membrane, respectively (**Figure 6A-C**). Raw g:Cost Functional Profiling data can be seen in **Supplementary Tables 4-5**.

The largest subcategories in upregulated LD-Aag2 genes were catalytic activity, membrane, intrinsic component of membrane, and integral component of membrane. These subcategories of genes were selected and further analyzed using the Functional Annotation Clustering tool at DAVID Bioinformatics Resources with the highest classification stringency. Upregulated LD-Aag2 genes associated with catalytic activity were grouped into 19 clusters (**Figure 7A**). Upregulated LD-Aag2 genes associated with membrane or intrinsic component of membrane were grouped into 20 clusters (**Figure 7B**). Genes associated with sugar and lipid transport and metabolism are represented.

Many differentially expressed genes were present that did not fall within a significantly altered functional gene category. We noted that some innate immunity genes associated with dengue virus (DENV) infection were also dysregulated in LD-Aag2 cells, including the gene DOME, which is a critical component of antiviral immunity (**Table 1**) [38-41]. We validated RNA-Seq data using TOLL5A and additional downregulated genes associated with the prophenoloxidase cascade 0, 12, and 24 hours post DENV infection. These data validated the RNA-Seq dataset, and also revealed that LD-Aag2 cells failed to induce TOLL5A expression during DENV infection (**Figure 8A-D**).

Table 1. Immunity gene expression during lipid depletion

Accession	LD vs. C[1]	Gene name	Source
AAEL002583	-0.69266	TOLL7	Li et al., 2020
AAEL007619	-2.95988	TOLL5A	Shin et al., 2006
AAEL000709	0.48146	CACT	Xi et al., 2008
AAEL007696	-0.13528	REL1A	Xi et al., 2008
AAEL001929	0.368022	SPZ5	Xi et al., 2008
AAEL015404	0.397152	LYSC	Xi et al., 2008
AAEL009178	1.840892	GNBPB4	Xi et al., 2008
AAEL006854	0.407618	ML	Xi et al., 2008
AAEL012267	0.643531	TEP13	Xi et al., 2008
AAEL001794	-5.71666	TEP20	Xi et al., 2008
AAEL005093	1.41003	CLIP46	Xi et al., 2008
AAEL007593	-0.51356	CLIPC2	Xi et al., 2008
AAEL000256	5.222971	SCRB9	Xi et al., 2008
AAEL014078	-2.30226	SRPN2	Xi et al., 2008
AAEL002730	-1.42432	SRPN21	Xi et al., 2008
AAEL009645	0.436617	Hypothetical	Xi et al., 2008
AAEL012471	-1.75139	DOME	Xi et al., 2008
AAEL012510	0.245783	IKK2	Xi et al., 2008
AAEL003439	-0.57157	CASPS18	Xi et al., 2008
AAEL012143	-0.45897	CASPS7	Xi et al., 2008
AAEL002309	0.275909	TPX4	Xi et al., 2008
AAEL000165	-0.23901	VAGO2	Russell et al., 2021
AAEL000200	1.159271	VAGO1	Russell et al., 2021
AAEL007624	-0.65794	REL2	Russell et al., 2021
AAEL007768	0.003417	MYD88	Russell et al., 2021

AAEL009496	0.185976	RPS7	Russell et al., 2021
AAEL010083	-0.64056	IMD	Russell et al., 2021
AAEL015527	-2.7863	Hypothetical	Russell et al., 2021
AAEL011763	-1.06255	PPO3	Ramirez et al., 2018
AAEL013501	-0.00116	PPO4	Ramirez et al., 2018
AAEL013492	-0.32532	PPO5	Ramirez et al., 2018
AAEL011764	0.947755	PPO10	Ramirez et al., 2018

[1]log2FoldChange. Differential gene expression over 50% is highlighted dark grey for downregulation and light grey for upregulation.

DENV infection in C and LD-Aag2 cells

Previous literature suggests that intracellular lipids are critical for DENV infection. LD-Aag2 cells were depleted of cholesterol and LD cell culture media had significantly less FFAs. We noted that LD-Aag2 responded to lipid-depleted conditions by upregulating gene pathways related to lipid metabolism and *de novo* lipid biosynthesis. Further, LD-Aag2 cells had lower cell proliferation, innate immunity genes are dysregulated, and a key innate immunity gene failed to respond to DENV infection. We hypothesized that chronic depletion of vertebrate lipids would influence DENV infection. To test this hypothesis, we inoculated monolayers of C-Aag2, LD-Aag2, and LD-6X-Aag2 cells with 50 focus forming units (FFUs) of DENV. Importantly, DENV was inoculated onto cells in LD cell culture media, and this media was replaced with either C, LD, or LD-6X cell culture media for the next three days. This avoided any influence of vertebrate lipids during the early stage of infection [8, 13]. We first determined when virus is actively being shed from C-Aag2 cells by collecting cell free supernatants 0, 1, 3, and 5 days post-infection (dpi) and quantifying viral RNA (vRNA). DENV shedding was first detected 3 dpi and shedding began to plateau 5 dpi (**Figure 9A**). We then collected cell free supernatants from virus producing cells 3 dpi and normalized vRNA to the number of virus-producing cells (**Figure 9B**). There was no difference in the amount of vRNA produced per cell. Normalizing vRNA to

cell number allowed us to control for differences in cell proliferation between cell types. We also took cell free supernatants from each cell type 3 dpi and inoculated fresh C-Aag2 monolayers. Freshly infected monolayers were incubated for three days and then fixed and stained with anti-DENV antibody. FFUs were quantified and normalized by the total number of virus producing cells. There was no significant difference in FFUs produced per cell (**Figure 9C-D**).

Discussion

The acquisition of dengue virus (DENV) in the mosquito is a complex process involving the virus and multiple vertebrate and invertebrate host factors. Our understanding of this process is ongoing and essential to develop acquisition and transmission-blocking strategies [1]. Our previous work revealed that vertebrate lipids modify an early stage of viral infection, and that blood meal derived low-density lipoprotein (LDL) and extracellular vesicles (EVs) enter mosquito cells and inhibit DENV membrane fusion [8, 13]. These studies suggest that reduced vertebrate lipids can enhance DENV infection and can increase the viral load in the mosquito. In nature, composition and frequency of blood meals will vary, which will lead some mosquitoes to experience nutrient deficiency and starvation [25-30]. In fact, experiments using an artificial diet showed that limiting dietary cholesterol can limit mosquito size and oogenesis [11]. To determine how *Ae. aegypti* and a mosquito-borne virus cope with depletion of vertebrate lipids, we developed an *in vitro* model system that consisted of feeding Aag2 cells with complete (C) and lipid-depleted (LD) cell culture media. In this context, C-Aag2 cells represent a blood fed mosquito that is replete with factors derived from vertebrate serum. LD-Aag2 cells represent mosquitoes that have not recently received a blood meal and are chronically depleted of vertebrate lipids including cholesterol and free fatty acids (FFAs). LD-6X-Aag2 cells are fed six

times glucose concentration and represent a mosquito that has access to plant nectar but has not recently received a blood meal. We also wanted to determine if excess glucose would be sufficient to recover the deficit in cell proliferation and lead to *de novo* lipid biosynthesis.

LD-Aag2 cells had a significantly lower level of cholesterol, but an equal amount of total intracellular lipids compared to C-Aag2. LD-Aag2 cells were slightly smaller in size and had their cell proliferation was reduced. Interestingly, LD-6X-Aag2 had increased levels of total intracellular lipids, suggesting that Aag2 cells can cope with chronic lipid depletion by inducing *de-novo* lipid biosynthesis. It was also evident that lipolysis of stored triglycerides abruptly stopped in C-Aag2 that were fed LD cell culture media. This suggests that Aag2 cells can sense a change in lipid availability and restrict consumption of lipids to survive nutrient deprivation. However, FFAs and TAGs were only mildly depleted in LD cell culture media. The fumed silica technique was more effective at removing cholesterol from fetal bovine serum. Considering that mosquitoes are cholesterol auxotrophs, it is possible that a change in cholesterol concentration is sensed by mosquito cells, which allows them to calibrate their metabolic needs. Cholesterol may act as a metabolic switch that communicates when a blood meal is available.

RNA-Seq revealed multiple pathways that were likely involved in deriving energy and producing invertebrate lipids. Genes associated with FFA metabolism were upregulated (e.g.,triacylglycerol lipase, diacylglycerol lipase, inactive pancreatic lipase-related protein 1, carboxylic ester hydrolase, and fatty acyl-CoA reductase), which likely led to catabolic breakdown of remaining FFA acids present in LD cell culture medium and production of ATP. We note that pyruvate carboxylase (AAEL009691), which is the rate-limiting enzyme responsible for fatty acid synthesis, was upregulated 2.5-fold. This, along with upregulation of

fatty acyl-CoA reductase supports that LD-Aag2 cells not only metabolize fatty acids for energy, but that they produce fatty acids *de novo* from precursor molecules. Genes associated with sugar hydrolysis and transport were also significantly upregulated, suggesting that LD-Aag2 cells relied heavily on sugar to maintain energy requirements. Genes related to production of extracellular matrix (ECM)-related proteins were also upregulated (e.g., immunoglobulin-like protein, fibronectin domain protein, and ECM protein), which may have been an adaptation to changes in the cell membrane and a need to express different proteins to facilitate adherence to the cell culture plate. LD-Aag2 cells down regulated a key gene related to gluconeogenesis (e.g., phosphoenolpyruvate carboxykinase), which likely conserved lipids and reduced cell proliferation. Phosphoenolpyruvate carboxykinase regulates triglyceride/fatty acid cycling, and downregulation suggests that lipids are being conserved in intracellular lipid droplets [42]. Fatty acid desaturases were also downregulated, suggesting that LD-Aag2 cells optimized lipid content by reducing double bonds and decreasing the space requirement per molecule. This would facilitate increased storage of intracellular lipids.

Previous studies have shown that flaviviruses reconfigure intracellular lipid membranes in host and mosquito cells, and cholesterol and lipid metabolism is manipulated during flavivirus infection [15-18, 20, 22, 36, 43, 44]. Recent literature has also shown that sugar feeding protects against arboviral infection in *Ae. aegypti*, and that vertebrate lipids can inhibit early stages of infection [8, 13, 14, 45]. Lipid metabolism and the nutritional status of a blood meal can clearly influence flavivirus infection; however, the role of cholesterol in invertebrate cells, which are cholesterol auxotrophs, is unclear [17, 46, 47].

Based on our previous observation that vertebrate lipids inhibit virus fusion, and that short-term depletion of vertebrate lipids from Aag2 cells inhibits infection [24], we hypothesized that DENV infection would be influenced by chronic depletion of vertebrate lipids. In contrast to our hypothesis, equal infectious units and vRNA were shed from C-Aag2, LD-Aag2, and LD-6X-Aag2 cells. This result may be explained by the conflicting nature of LD-Aag2 cells. On one hand, LD-Aag2 cells should undermine DENV infection because of the lack of cholesterol and their low energy and mitotic state. However, key innate immunity genes were downregulated in LD-Aag2 cells, and TOLL5A failed to respond to infection. This dichotomy may allow for DENV to persist in mosquitoes despite a fluctuation in vertebrate lipids and nutrition. These data highlight the adaptability of both mosquitoes and DENV. Aag2 cells were able to adapt to lipid depletion by leveraging sugar and lipid metabolic pathways, and DENV was able to replicate to the same degree regardless of nutritional differences perhaps due to utilization of invertebrate-specific lipids and/or suppressed innate immunity. This is not entirely surprising given the challenges mosquitoes face in acquiring a blood meal and their ability to survive on plant nectar and sugar alone. It is also not surprising given that the DENV life cycle requires transmission between invertebrate and vertebrate species.

We also considered the reduced cell size and proliferation in LD-Aag2 cells and hypothesized that this was due to the loss in vertebrate lipids as an energy source. Despite a significant reduction in certain lipid species, it was possible that remaining fatty acids present in fetal bovine serum (FBS) may provide chemical energy via lipolysis. Interestingly, C-Aag2 and LD-Aag2 cells contained the same levels of intracellular ATP. These data suggest that LD-Aag2 cells may use glucose and FFAs present in LD cell culture media for energy production and that

the reduced cell size and mitotic rate may due to factors other than low levels of ATP. One possibility is that vertebrate sterols serve as structural components for cell membranes and lipid containing organelles, and that deprivation of these raw materials limits cell growth and replication [17, 23, 24, 46, 48]. Previous research showed that small mosquitoes have less cholesterol and glycerol stores and that cholesterol depletion reduces egg production [11]. Our previous research showed that both free cholesterol and cholesterol in the form of LDL can rapidly enter into mosquito cells and integrate into mosquito cell membranes *in vitro* and *in vivo* and other studies support that sterols are shuttled into the insect cells and incorporated into membranes [8, 17, 23]. It is also possible that vertebrate lipids such as cholesterol integrate into lipid rafts along with sphingomyelin and provide a cell signaling function, which may promote cell growth and replication [14, 49-51].

Determining how virus-vector-host interactions impact virus acquisition and transmission will reveal novel strategies to mitigate vector-borne disease. This study revealed that *Ae. aegypti* adapts to lipid depletion by reducing lipolysis and inducing gene pathways related to sugar and lipid metabolism, including *de novo* lipid biosynthesis. It also revealed that DENV replicates equally well when vertebrate lipids are depleted from mosquito cells. This is an important investigation because mosquitoes will contain different concentrations of host material depending on their blood meal history, and individual hosts will have significantly different concentrations of certain lipids in their blood. These data reveal that mosquito cells may use vertebrate lipids like cholesterol to sense their environment and shift metabolic pathways to make use of vertebrate factors and to cope during times where a blood meal is not available.

Future research is needed to determine if it is possible to target lipid metabolism as a method to control mosquito populations and the pathogens that they carry.

Materials and Methods

Cell culture and virus production

Ae. aegypti Aag2 were a kind gift from Erol Fikrig and were grown at 27 °C with 5% CO_2 in either complete (C), lipid-depleted (LD), or LD with 6X glucose (LD-6X) cell culture media. Complete cell culture media contained high glucose DMEM (Gibco) media supplemented with 10% heat-inactivated fetal bovine serum (FBS) (Gemini, CA), 1% penicillin-streptomycin (Gibco), and 1% tryptose phosphate broth (Sigma, MO). Cells fed complete (C) cell culture media are listed as C-Aag2 cells. LD serum was made by incubating FBS with fumed silica (Millipore Sigma) overnight followed by removal of silica-lipid complexes by centrifugation according to previously published protocols [24, 34, 35]. LD-Aag2 cells represent C-Aag2 cells that were fed complete (LD) cell culture media every other day for three months. LD-Aag2 cells began to expand after two months of feeding with LD cell culture media, and stocks of passage zero cells were frozen at -80°C. Both C-Aag2 and LD-Aag2 cells are fed with cell culture media that contains 4.5 g/L D-glucose, while LD-6X media contained 27 g/L D-glucose. Digital images of cell morphology were taken using an EVOS XL Core Imaging System at 20X magnification. Cell proliferation assays were performed after seeding 100 cells/well in triplicate in 96 well plates, followed by hemocytometer counts for 7 days. Cells were fed with either C or LD cell culture media every other day throughout the time course. *Ae. albopictus* C6/36 cells and dengue virus type 2 (DENV2) New Guinea C were a kind gift from Erol Fikrig. C6/36 cells were

infected with DENV2 at a MOI of 1.0 and cell-free virus stocks were harvested 7 days post-infection (dpi) and stored at −80 °C until use (36).

Cholesterol, Free fatty acid, Triacylglycerol, and Glycerol assays

Cholesterol was measured using the Amplex Red Cholesterol Assay Kit (Invitrogen). Cell culture media and Aag2 cells that were treated with either C or LD cell culture medium for 1-7 days were lysed in PBS containing 1% Triton X-100 and protease inhibitor cocktail and used for analysis. Equivalent volumes of cell culture medium or 2 ug of total protein was used for each sample. The reactions were brought up to a total reaction volume of 50 ul using 1x reaction buffer from the kit and applied to individual wells on a 96-well microplate. 50 uL of Amplex Red reagent/HRP/cholesterol oxidase/cholesterol esterase working solution was added to each well and the plate was incubated at 37 °C for 30 minutes protected from light. Fluorescence was measured using an Epoch2 microplate spectrophotometer and Gen5 software (Agilent) with excitation at 550nm and emission detection at 590nm [24]. Free fatty acids (FFAs) were quantified in C and LD cell culture media using the Free Fatty Acid Fluorometric Assay Kit according to manufacturer's instructions (Cayman Chemical). Triacylglycerols (TAGs) were quantified in C and LD cell culture media using the Triglyceride Colorimetric Assay Kit according to manufacturer's instructions (Cayman Chemical). Glycerol was quantified in C-Aag2 cell free supernatants after treatment with C and LD cell culture media 1, 3, 5, and 7 days

post-treatment using the Glycerol Colorimetric Assay Kit according to manufacturer's instructions (Cayman Chemical). Each sample of cell culture media or cell free lysate was measured in triplicate using the Epoch2 microplate spectrophotometer and Gen5 software (Agilent).

Nile Red Total Intracellular Lipid Assay

The Nile Red lipid droplet stain was obtained from Santa Cruz Biotechnology. The Hoescht nuclei counterstain was obtained from Life Technologies. Total intracellular lipids were stained using Nile Red and the Hoescht stain was used to quantify and normalize data by cell number as needed [37]. Briefly, 100,000 C, LD, and LD-6X-Aag2 cells were seeded in a 96 well plate in triplicate for 48 hours. Cell were then fixed with 4% paraformaldehyde in PBS for 10 minutes before staining cells with Hoescht (1 µg/mL in PBS) for 15 minutes, and Nile Red (1 µg/mL in PBS) for 15 minutes. Ten representative digital photos were taken using an EVOS fluorescent microscope at 20X magnification across the triplicate samples and photos were analyzed using ImageJ software. Nile Red staining was used to measure the relative cell area of C, LD, and LD-6X-Aag2 cells, and Nile Red pixel intensity coupled with Hoescht staining was used to measure the relative amount of total intracellular lipids per cell.

ATP Assay

The Colorimetric ATP Assay kit was purchased from BioVision and used with Vivaspin 500 10kDa MWCO spin filters purchased from GE Healthcare. Briefly, C and LD-Aag2 cells were grown to confluence in 12-well plates in triplicate. This assay was conducted 3 days post feeding of each cell line with their respective insect cell growth medium. 17,000 cells of each cell line were pelleted at 1,700 RPM for 7 minutes, and then re-suspended in ATP lysis buffer. Cell lysates were then passed through 10kDa MWCO spin filters before following manufacturer's instructions. Each respective cell lysate sample was measured in duplicate at OD-570 nm using the Epoch2 microplate spectrophotometer and Gen5 software (Agilent).

RNA Sequencing (RNASeq)

RNA Sequencing (RNASeq) was conducted using a service offered by Novogene, USA (California UC Davis) on C and LD-Aag2 cells in triplicate. RNA was extracted using the Qiagen RNeasy Plus RNA Extraction kit with gDNA eliminator columns. RNA integrity was assessed using the RNA Nano 6000 Assay Kit of the Bioanalyzer 2100 system (Agilent Technologies, CA, USA). Total RNA was used as input material for the RNA sample preparations. Briefly, mRNA was purified from total RNA using poly-T oligo-attached magnetic beads. Fragmentation was carried out using divalent cations under elevated temperature in First Strand Synthesis Reaction Buffer(5X). First strand cDNA was synthesized using random hexamer primer and M-MuLV Reverse Transcriptase(RNase H-). Second strand cDNA synthesis was subsequently performed using DNA Polymerase I and RNase H. Remaining overhangs were converted into blunt ends via exonuclease/polymerase activities. After adenylation of 3' ends of DNA fragments, Adaptor with hairpin loop structure were ligated to prepare for hybridization. In order to select cDNA fragments of preferentially 370~420 bp in length, the library fragments

were purified with AMPure XP system (Beckman Coulter, Beverly, USA). Then PCR was performed with Phusion High-Fidelity DNA polymerase, Universal PCR primers and Index (X) Primer. At last, PCR products were purified (AMPure XP system) and library quality was assessed on the Agilent Bioanalyzer 2100 system. The clustering of the index-coded samples was performed on a cBot Cluster Generation System using TruSeq PE Cluster Kit v3-cBot-HS (Illumia) according to the manufacturer’s instructions. After cluster generation, the library preparations were sequenced on an Illumina Novaseq platform and 150 bp paired-end reads were generated. Raw data (raw reads) of fastq format were firstly processed through in-house perl scripts. In this step, clean data (clean reads) were obtained by removing reads containing adapter, reads containing ploy-N and low-quality reads from raw data. At the same time, Q20, Q30 and GC content the clean data were calculated. All the downstream analyses were based on the clean data with high quality. Reference genome and gene model annotation files were downloaded from genome website directly. Index of the reference genome was built using Hisat2 v2.0.5 and paired-end clean reads were aligned to the reference genome using Hisat2 v2.0.5. We selected Hisat2 as the mapping tool for that Hisat2 can generate a database of splice junctions based on the gene model annotation file and thus a better mapping result than other non-splice mapping tools. featureCounts v1.5.0-p3 was used to count the reads numbers mapped to each gene. FPKM of each gene was calculated based on the length of the gene and reads count mapped to this gene. FPKM, expected number of Fragments Per Kilobase of transcript sequence per Millions base pairs sequenced, considers the effect of sequencing depth and gene length for the reads count at the same time, and is currently the most commonly used method for estimating gene expression levels. Differential expression analysis of the two

conditions/groups was performed using the DESeq2 R package (1.20.0). DESeq2 provide statistical routines for determining differential expression in digital gene expression data using a model based on the negative binomial distribution. The resulting P-values were adjusted using the Benjamini and Hochberg's approach for controlling the false discovery rate . Genes with an adjusted P-value <=0.05 found by DESeq2 were assigned as differentially expressed. Functional annotations of differentially expressed genes were performed using the g:Cost Functional Profiling tool at g:Profiler and NCBI's DAVID Functional Annotation Bioinformatics tool.

Gene expression validation and DENV2 vRNA RT-qPCR assays

For gene expression validation, C and LD-Aag2 cells were grown to ~70% confluence in 96 well plates and inoculated with 50 focus-forming units (FFUs) of DENV2 in LD cell culture media for 1 hour at 27 °C in triplicate. Inoculation of DENV2 in LD cell culture media is important in order to avoid inhibiting virus fusion with vertebrate extracellular vesicles [24]. Unbound virus was removed and fresh C and LD cell culture media was added onto their respective cell type. Total cellular RNA was extracted at 0, 12, and 24 hours post infection (hpi) using an RNeasy RNA Extraction kit with gRNA eliminator columns (Qiagen). Total RNA was analyzed using a singleplex format in 48-well plates with a total reaction volume of 10 µl using an Eco Illumina instrument. Reverse transcription and quantitative PCR were performed in the same closed tube with 50 ng of total RNA per reaction using the Power SYBR Green RNA-to-Ct 1-Step RT-qPCR Kit (ThermoFisher). All primers were used at a final concentration of 1 µM. Primer sequences are available in **Supplemental Table 6**. Cycling conditions were 50 °C for 30 min (reverse transcription) and 95 °C for 15 min, followed by 45 cycles of 94 °C for 15 s, 55 °C for 30 s and 72 °C for 30 s. Fold-differences in gene expression were determined using the Pfaffl

method and data were normalized based on total nanograms of cellular RNA. For DENV2 viral RNA (vRNA) analysis, C, LD, and LD-6X-Aag2 cells were grown to ~70% confluence in 96 well plates and inoculated with 50 focus-forming units (FFUs) of DENV2 in LD cell culture media for 1 hour at 27 °C in triplicate. Unbound virus was removed and fresh C, LD, and LD-6X cell culture media was added onto their respective cell type. At 3 dpi, 10 µL of cell free supernatants were harvested and spiked into RLT buffer from the RNeasy RNA extraction kit (Qiagen). At this time, the total number of cells per well were quantified using a hemocytometer. This was performed to account for any differences in cell proliferation between cell types. RNA was then extraction as listed above, following by RT-qPCR using F 5′ CAG ATC TCT GAT GAA TAA CCA ACG 3 and R 5′ CAT TCC AAG TGA GAA TCC TTT GTC A 3’primers. The same protocol as shown above was used for DENV2 vRNA RT-qPCR, and relative vRNA levels were normalized per cell.

Focus Forming Unit (FFU) infectivity assays

C, LD, and LD-6X-Aag2 cells were grown to ~70% confluence in 96 well plates and inoculated with 50 focus-forming units (FFUs) of DENV2 in LD cell culture media for 1 hour at 27 °C. Inoculation of DENV2 in LD cell culture media is important in order to avoid inhibiting virus fusion with vertebrate extracellular vesicles [24]. Unbound virus was removed and fresh C, LD, and LD-6X-Aag2 cell culture media was added onto their respective cell type. At 3 dpi, 70 uL of C, LD, and LD-6X- Aag2 cell-free supernatants were inoculated onto fresh ~70% confluence monolayers of C-Aag2 cells. Unbound virus was removed after 1 hour and fresh C cell culture media was added onto each of the monolayers. At 3 dpi, confluent C-Aag2 monolayers were fixed in 4% paraformaldehyde in phosphate buffered saline (PBS) for 10

minutes, cell membranes were permeabilized with 0.1% Triton X-100, and DENV2 antigen was stained with 1:200 anti-DENV2 envelope antibody (3H5-1, EMD Millipore) in PBS with 1% bovine serum albumin (BSA). The total number of DENV-positive foci were revealed using a 1:200 horseradish peroxidase-conjugated secondary antibody in PBS plus 1% BSA, and an AEC Peroxidase Substrate kit (Vector Laboratories). DENV2-positive foci were quantified using the Evos XL Core Imaging System. DENV2 FFUs were normalized to the total number of producing C, LD, and LD-6X-Aag2 cells present 3 dpi. The total number of producing cells were quantified using a hemocytometer after cell free supernatants were harvested. This was performed to account for any differences in cell proliferation between cell types.

REFERENCES CITED

1. Conway MJ, Colpitts TM, Fikrig E. Role of the Vector in Arbovirus Transmission. Annu Rev Virol. 2014;1(1):71-88. Epub 2014/11/03. doi: 10.1146/annurev-virology-031413-085513 [doi]. PubMed PMID: 26958715.
2. Gould EA, Solomon T. Pathogenic flaviviruses. Lancet. 2008;371(9611):500-9. Epub 2008/02/12. doi: S0140-6736(08)60238-X [pii]
10.1016/S0140-6736(08)60238-X [doi]. PubMed PMID: 18262042.
3. Guzman MG, Alvarez M, Halstead SB. Secondary infection as a risk factor for dengue hemorrhagic fever/dengue shock syndrome: an historical perspective and role of antibody-dependent enhancement of infection. Arch Virol. 2013;158(7):1445-59. Epub 2013/03/09. doi: 10.1007/s00705-013-1645-3 [doi]. PubMed PMID: 23471635.
4. Aguiar MF, Halstead SB. Dengue Vaccines: are they safe for Travelers? Travel Med Infect Dis. 2016. Epub 2016/06/28. doi: S1477-8939(16)30071-0 [pii]
10.1016/j.tmaid.2016.06.005 [doi]. PubMed PMID: 27343438.
5. Deng SQ, Yang X, Wei Y, Chen JT, Wang XJ, Peng HJ. A Review on Dengue Vaccine Development. Vaccines (Basel). 2020;8(1). Epub 2020/02/07. doi: vaccines8010063 [pii]
vaccines-08-00063 [pii]
10.3390/vaccines8010063 [doi]. PubMed PMID: 32024238; PubMed Central PMCID: PMC7159032.
6. Tsheten T, Clements ACA, Gray DJ, Adhikary RK, Wangdi K. Clinical features and outcomes of COVID-19 and dengue co-infection: a systematic review. BMC Infect Dis. 2021;21(1):729. Epub 2021/08/04. doi: 10.1186/s12879-021-06409-9 [pii]
6409 [pii]

10.1186/s12879-021-06409-9 [doi]. PubMed PMID: 34340682; PubMed Central PMCID: PMC8327042.

7. Manning JE, Morens DM, Kamhawi S, Valenzuela JG, Memoli M. Mosquito Saliva: The Hope for a Universal Arbovirus Vaccine? J Infect Dis. 2018;218(1):7-15. Epub 2018/04/05. doi: 4956143 [pii] jiy179 [pii] 10.1093/infdis/jiy179 [doi]. PubMed PMID: 29617849; PubMed Central PMCID: PMC5989627.

8. Wagar ZL, Tree MO, Mpoy MC, Conway MJ. Low density lipopolyprotein inhibits flavivirus acquisition in Aedes aegypti. Insect Mol Biol. 2017. Epub 2017/07/19. doi: 10.1111/imb.12334 [doi]. PubMed PMID: 28718976.

9. Ma E, Zhu Y, Liu Z, Wei T, Wang P, Cheng G. Interaction of Viruses with the Insect Intestine. Annu Rev Virol. 2021;8(1):115-31. Epub 2021/04/20. doi: 10.1146/annurev-virology-091919-100543 [doi]. PubMed PMID: 33872516.

10. Erlandson MA, Toprak U, Hegedus DD. Role of the peritrophic matrix in insect-pathogen interactions. J Insect Physiol. 2019;117:103894. Epub 2019/06/09. doi: S0022-1910(19)30030-7 [pii] 10.1016/j.jinsphys.2019.103894 [doi]. PubMed PMID: 31175854.

11. Talyuli OA, Bottino-Rojas V, Taracena ML, Soares AL, Oliveira JH, Oliveira PL. The use of a chemically defined artificial diet as a tool to study Aedes aegypti physiology. J Insect Physiol. 2015;83:1-7. Epub 2015/11/19. doi: S0022-1910(15)00217-6 [pii] 10.1016/j.jinsphys.2015.11.007 [doi]. PubMed PMID: 26578294.

12. Kogan PH. Substitute blood meal for investigating and maintaining Aedes aegypti (Diptera: Culicidae). J Med Entomol. 1990;27(4):709-12. Epub 1990/07/01. doi: 10.1093/jmedent/27.4.709 [doi]. PubMed PMID: 2388248.

13. Freitas MN, Marten AD, Moore GA, Tree MO, McBrayer SP, Conway MJ. Extracellular vesicles restrict dengue virus fusion in Aedes aegypti cells. Virology. 2020;541:141-9. Epub 2020/02/15. doi: S0042-6822(19)30364-2 [pii] 10.1016/j.virol.2019.12.010 [doi]. PubMed PMID: 32056712.

14. Lee CJ, Lin HR, Liao CL, Lin YL. Cholesterol effectively blocks entry of flavivirus. J Virol. 2008;82(13):6470-80. Epub 2008/05/02. doi: JVI.00117-08 [pii] 10.1128/JVI.00117-08 [doi]. PubMed PMID: 18448543; PubMed Central PMCID: PMC2447114.

15. Akey DL, Brown WC, Dutta S, Konwerski J, Jose J, Jurkiw TJ, et al. Flavivirus NS1 structures reveal surfaces for associations with membranes and the immune system. Science. 2014;343(6173):881-5. Epub 2014/02/08. doi: science.1247749 [pii] 10.1126/science.1247749 [doi]. PubMed PMID: 24505133; PubMed Central PMCID: PMC4263348.

16. Chotiwan N, Andre BG, Sanchez-Vargas I, Islam MN, Grabowski JM, Hopf-Jannasch A, et al. Dynamic remodeling of lipids coincides with dengue virus replication in the midgut of Aedes aegypti mosquitoes. PLoS Pathog. 2018;14(2):e1006853. Epub 2018/02/16. doi: 10.1371/journal.ppat.1006853 [doi] PPATHOGENS-D-16-00829 [pii]. PubMed PMID: 29447265; PubMed Central PMCID: PMC5814098.

17. Fu Q, Inankur B, Yin J, Striker R, Lan Q. Sterol Carrier Protein 2, a Critical Host Factor for Dengue Virus Infection, Alters the Cholesterol Distribution in Mosquito Aag2 Cells. J Med Entomol. 2015;52(5):1124-34. Epub 2015/09/04. doi: tjv101 [pii]

10.1093/jme/tjv101 [doi]. PubMed PMID: 26336241.

18. Geoghegan V, Stainton K, Rainey SM, Ant TH, Dowle AA, Larson T, et al. Perturbed cholesterol and vesicular trafficking associated with dengue blocking in Wolbachia-infected Aedes aegypti cells. Nat Commun. 2017;8(1):526. Epub 2017/09/15. doi: 10.1038/s41467-017-00610-8 [doi] 10.1038/s41467-017-00610-8 [pii]. PubMed PMID: 28904344; PubMed Central PMCID: PMC5597582.

19. Lee CJ, Liao CL, Lin YL. Flavivirus activates phosphatidylinositol 3-kinase signaling to block caspase-dependent apoptotic cell death at the early stage of virus infection. J Virol. 2005;79(13):8388-99. Epub 2005/06/16. doi: 79/13/8388 [pii] 10.1128/JVI.79.13.8388-8399.2005 [doi]. PubMed PMID: 15956583; PubMed Central PMCID: PMC1143730.

20. Molloy JC, Sommer U, Viant MR, Sinkins SP. Wolbachia Modulates Lipid Metabolism in Aedes albopictus Mosquito Cells. Appl Environ Microbiol. 2016;82(10):3109-20. Epub 2016/03/20. doi: AEM.00275-16 [pii] 00275-16 [pii] 10.1128/AEM.00275-16 [doi]. PubMed PMID: 26994075; PubMed Central PMCID: PMC4959074.

21. Soto-Acosta R, Mosso C, Cervantes-Salazar M, Puerta-Guardo H, Medina F, Favari L, et al. The increase in cholesterol levels at early stages after dengue virus infection correlates with an augment in LDL particle uptake and HMG-CoA reductase activity. Virology. 2013;442(2):132-47. Epub 2013/05/07. doi: S0042-6822(13)00197-9 [pii] 10.1016/j.virol.2013.04.003 [doi]. PubMed PMID: 23642566.

22. Zaitseva E, Yang ST, Melikov K, Pourmal S, Chernomordik LV. Dengue virus ensures its fusion in late endosomes using compartment-specific lipids. PLoS Pathog. 2010;6(10):e1001131. Epub 2010/10/16. doi: 10.1371/journal.ppat.1001131 [doi]. PubMed PMID: 20949067; PubMed Central PMCID: PMC2951369.

23. Lan Q, Massey RJ. Subcellular localization of the mosquito sterol carrier protein-2 and sterol carrier protein-x. J Lipid Res. 2004;45(8):1468-74. Epub 2004/05/18. doi: S0022-2275(20)31308-0 [pii] 10.1194/jlr.M400003-JLR200 [doi]. PubMed PMID: 15145982.

24. Tree MO, Londono-Renteria B, Troupin A, Clark KM, Colpitts TM, Conway MJ. Dengue virus reduces expression of low-density lipoprotein receptor-related protein 1 to facilitate replication in Aedes aegypti. Sci Rep. 2019;9(1):6352. Epub 2019/04/25. doi: 10.1038/s41598-019-42803-9 [doi] 10.1038/s41598-019-42803-9 [pii]. PubMed PMID: 31015516; PubMed Central PMCID: PMC6478881.

25. Norris EJ, Bloomquist JR. Nutritional status significantly affects toxicological endpoints in the CDC bottle bioassay. Pest Manag Sci. 2022;78(2):743-8. Epub 2021/10/26. doi: 10.1002/ps.6687 [doi]. PubMed PMID: 34693614.

26. Ross PA, Endersby NM, Hoffmann AA. Costs of Three Wolbachia Infections on the Survival of Aedes aegypti Larvae under Starvation Conditions. PLoS Negl Trop Dis. 2016;10(1):e0004320. Epub 2016/01/09. doi: PNTD-D-15-01787 [pii] 10.1371/journal.pntd.0004320 [doi]. PubMed PMID: 26745630; PubMed Central PMCID: PMC4706305.

27. Price DP, Schilkey FD, Ulanov A, Hansen IA. Small mosquitoes, large implications: crowding and starvation affects gene expression and nutrient accumulation in Aedes aegypti. Parasit Vectors. 2015;8:252. Epub 2015/05/01. doi: 10.1186/s13071-015-0863-9 [pii]
863 [pii]
10.1186/s13071-015-0863-9 [doi]. PubMed PMID: 25924822; PubMed Central PMCID: PMC4415286.

28. Muturi EJ, Kim CH, Alto BW, Berenbaum MR, Schuler MA. Larval environmental stress alters Aedes aegypti competence for Sindbis virus. Trop Med Int Health. 2011;16(8):955-64. Epub 2011/05/14. doi: 10.1111/j.1365-3156.2011.02796.x [doi]. PubMed PMID: 21564427.

29. Canyon DV, Hii JL, Muller R. Effect of diet on biting, oviposition, and survival of Aedes aegypti (Diptera: Culicidae). J Med Entomol. 1999;36(3):301-8. Epub 1999/05/25. doi: 10.1093/jmedent/36.3.301 [doi]. PubMed PMID: 10337099.

30. Scott TW, Naksathit A, Day JF, Kittayapong P, Edman JD. A fitness advantage for Aedes aegypti and the viruses it transmits when females feed only on human blood. Am J Trop Med Hyg. 1997;57(2):235-9. Epub 1997/08/01. doi: 10.4269/ajtmh.1997.57.235 [doi]. PubMed PMID: 9288822.

31. Stollar V, Thomas VL. An agent in the Aedes aegypti cell line (Peleg) which causes fusion of Aedes albopictus cells. Virology. 1975;64(2):367-77. Epub 1975/04/01. doi: 10.1016/0042-6822(75)90113-0 [doi]. PubMed PMID: 806166.

32. Walker T, Jeffries CL, Mansfield KL, Johnson N. Mosquito cell lines: history, isolation, availability and application to assess the threat of arboviral transmission in the United Kingdom. Parasit Vectors. 2014;7:382. Epub 2014/08/22. doi: 1756-3305-7-382 [pii]
1557 [pii]
10.1186/1756-3305-7-382 [doi]. PubMed PMID: 25141888; PubMed Central PMCID: PMC4150944.

33. Weger-Lucarelli J, Rückert C, Grubaugh ND, Misencik MJ, Armstrong PM, Stenglein MD, et al. Adventitious viruses persistently infect three commonly used mosquito cell lines. Virology. 2018;521:175-80. Epub 2018/06/30. doi: S0042-6822(18)30184-3 [pii]
10.1016/j.virol.2018.06.007 [doi]. PubMed PMID: 29957338; PubMed Central PMCID: PMC6764424.

34. Brovkovych V, Aldrich A, Li N, Atilla-Gokcumen GE, Frasor J. Removal of Serum Lipids and Lipid-Derived Metabolites to Investigate Breast Cancer Cell Biology. Proteomics. 2019;19(18):e1800370. Epub 2019/03/29. doi: 10.1002/pmic.201800370 [doi]. PubMed PMID: 30919589; PubMed Central PMCID: PMC6742575.

35. Ferraz TP, Fiuza MC, Dos Santos ML, Pontes De Carvalho L, Soares NM. Comparison of six methods for the extraction of lipids from serum in terms of effectiveness and protein preservation. J Biochem Biophys Methods. 2004;58(3):187-93. Epub 2004/03/18. doi: 10.1016/j.jbbm.2003.10.008 [doi]
S0165022X03002057 [pii]. PubMed PMID: 15026205.

36. O'Neal AJ, Butler LR, Rolandelli A, Gilk SD, Pedra JH. Lipid hijacking: a unifying theme in vector-borne diseases. Elife. 2020;9. Epub 2020/10/30. doi: 61675 [pii]
10.7554/eLife.61675 [doi]. PubMed PMID: 33118933; PubMed Central PMCID: PMC7595734.

37. Greenspan P, Mayer EP, Fowler SD. Nile red: a selective fluorescent stain for intracellular lipid droplets. J Cell Biol. 1985;100(3):965-73. Epub 1985/03/01. doi: 85131346 [pii]
10.1083/jcb.100.3.965 [doi]. PubMed PMID: 3972906; PubMed Central PMCID: PMC2113505.

38. Xi Z, Ramirez JL, Dimopoulos G. The Aedes aegypti toll pathway controls dengue virus infection. PLoS Pathog. 2008;4(7):e1000098. Epub 2008/07/08. doi: 08-PLPA-RA-0161R2 [pii]
10.1371/journal.ppat.1000098 [doi]. PubMed PMID: 18604274; PubMed Central PMCID: PMC2435278.
39. Russell TA, Ayaz A, Davidson AD, Fernandez-Sesma A, Maringer K. Imd pathway-specific immune assays reveal NF-κB stimulation by viral RNA PAMPs in Aedes aegypti Aag2 cells. PLoS Negl Trop Dis. 2021;15(2):e0008524. Epub 2021/02/17. doi: PNTD-D-20-01135 [pii]
10.1371/journal.pntd.0008524 [doi]. PubMed PMID: 33591970; PubMed Central PMCID: PMC7909628.
40. Ramirez JL, Muturi EJ, Dunlap C, Rooney AP. Strain-specific pathogenicity and subversion of phenoloxidase activity in the mosquito Aedes aegypti by members of the fungal entomopathogenic genus Isaria. Sci Rep. 2018;8(1):9896. Epub 2018/07/04. doi: 10.1038/s41598-018-28210-6 [pii]
28210 [pii]
10.1038/s41598-018-28210-6 [doi]. PubMed PMID: 29967469; PubMed Central PMCID: PMC6028645.
41. Rodriguez-Andres J, Rani S, Varjak M, Chase-Topping ME, Beck MH, Ferguson MC, et al. Phenoloxidase activity acts as a mosquito innate immune response against infection with Semliki Forest virus. PLoS Pathog. 2012;8(11):e1002977. Epub 2012/11/13. doi: 10.1371/journal.ppat.1002977 [doi]
PPATHOGENS-D-12-01166 [pii]. PubMed PMID: 23144608; PubMed Central PMCID: PMC3493465.
42. Millward CA, Desantis D, Hsieh CW, Heaney JD, Pisano S, Olswang Y, et al. Phosphoenolpyruvate carboxykinase (Pck1) helps regulate the triglyceride/fatty acid cycle and development of insulin resistance in mice. J Lipid Res. 2010;51(6):1452-63. Epub 2010/02/04. doi: S0022-2275(20)41019-3 [pii]
m005363 [pii]
10.1194/jlr.M005363 [doi]. PubMed PMID: 20124556; PubMed Central PMCID: PMC3035508.
43. Cheon HM, Shin SW, Bian G, Park JH, Raikhel AS. Regulation of lipid metabolism genes, lipid carrier protein lipophorin, and its receptor during immune challenge in the mosquito Aedes aegypti. J Biol Chem. 2006;281(13):8426-35. Epub 2006/02/02. doi: M510957200 [pii]
10.1074/jbc.M510957200 [doi]. PubMed PMID: 16449228.
44. Biswas HH, Gordon A, Nunez A, Perez MA, Balmaseda A, Harris E. Lower Low-Density Lipoprotein Cholesterol Levels Are Associated with Severe Dengue Outcome. PLoS Negl Trop Dis. 2015;9(9):e0003904. Epub 2015/09/04. doi: 10.1371/journal.pntd.0003904 [doi]
PNTD-D-14-01837 [pii]. PubMed PMID: 26334914; PubMed Central PMCID: PMC4559460.
45. Almire F, Terhzaz S, Terry S, McFarlane M, Gestuveo RJ, Szemiel AM, et al. Sugar feeding protects against arboviral infection by enhancing gut immunity in the mosquito vector Aedes aegypti. PLoS Pathog. 2021;17(9):e1009870. Epub 2021/09/03. doi: PPATHOGENS-D-21-00183 [pii]
10.1371/journal.ppat.1009870 [doi]. PubMed PMID: 34473801; PubMed Central PMCID: PMC8412342.
46. Hafer A, Whittlesey R, Brown DT, Hernandez R. Differential incorporation of cholesterol by Sindbis virus grown in mammalian or insect cells. J Virol. 2009;83(18):9113-21. Epub 2009/07/10. doi: JVI.00755-09 [pii]

0755-09 [pii]
10.1128/JVI.00755-09 [doi]. PubMed PMID: 19587056; PubMed Central PMCID: PMC2738221.

47. Sousa IP, Jr., Carvalho CA, Ferreira DF, Weissmüller G, Rocha GM, Silva JL, et al. Envelope lipid-packing as a critical factor for the biological activity and stability of alphavirus particles isolated from mammalian and mosquito cells. J Biol Chem. 2011;286(3):1730-6. Epub 2010/11/16. doi: S0021-9258(20)56205-4 [pii]
M110.198002 [pii]
10.1074/jbc.M110.198002 [doi]. PubMed PMID: 21075845; PubMed Central PMCID: PMC3023467.

48. Ahn A, Schoepp RJ, Sternberg D, Kielian M. Growth and stability of a cholesterol-independent Semliki Forest virus mutant in mosquitoes. Virology. 1999;262(2):452-6. Epub 1999/09/30. doi: S0042-6822(99)99932-4 [pii]
10.1006/viro.1999.9932 [doi]. PubMed PMID: 10502523.

49. von Tresckow B, Kallen KJ, von Strandmann EP, Borchmann P, Lange H, Engert A, et al. Depletion of cellular cholesterol and lipid rafts increases shedding of CD30. J Immunol. 2004;172(7):4324-31. Epub 2004/03/23. PubMed PMID: 15034047.

50. Ouweneel AB, Thomas MJ, Sorci-Thomas MG. The ins and outs of lipid rafts: functions in intracellular cholesterol homeostasis, microparticles, and cell membranes: Thematic Review Series: Biology of Lipid Rafts. J Lipid Res. 2020;61(5):676-86. Epub 2021/03/16. doi: S0022-2275(20)43617-X [pii]
tR119000383 [pii]
10.1194/jlr.TR119000383 [doi]. PubMed PMID: 33715815; PubMed Central PMCID: PMC7193959.

51. Ando J, Kinoshita M, Cui J, Yamakoshi H, Dodo K, Fujita K, et al. Sphingomyelin distribution in lipid rafts of artificial monolayer membranes visualized by Raman microscopy. Proc Natl Acad Sci U S A. 2015;112(15):4558-63. Epub 2015/04/01. doi: 1418088112 [pii]
201418088 [pii]
10.1073/pnas.1418088112 [doi]. PubMed PMID: 25825736; PubMed Central PMCID: PMC4403184.

Supplemental Table 1: Complete readcount and FPKM list of genes in C vs LD-Aag2 cells

Supplemental Table 2: Complete readcount and FPKM list of upregulated genes in C vs LD-Aag2 cells

Supplemental Table 3: Complete readcount and FPKM list of downregulated genes in C vs LD-Aag2 cells

Supplemental Table 4: g:Cost functional profiling analysis of upregulated LD-Aag2 genes

Supplemental Table 5: g:Cost functional profiling analysis of downregulated LD-Aag2 genes

Supplemental Table 6: Primers sequences for RT-qPCR

Figure legends

Figure 1: Lipid-depleted cell culture media has less cholesterol, free fatty acids, and triacylglycerol, but can support replication of LD-Aag2 cells.

(A) Amplex Red cholesterol assay of complete (C) and lipid-depleted (LD) cell culture media. (**B)** Amplex Red cholesterol assay of C-Aag2 and LD-Aag2 cell lysates at days 1, 2, 3, and 7 post-treatment with C and LD cell culture media. **(C)** Free fatty acid (FFA) assay of complete C and LD cell culture media. **(D)** Triacylglycerol assay of C and LD cell culture media. (**E)** Representative images of C-Aag2 and LD-Aag2 cells. **(F)** Cell proliferation assay of C-Aag2 (circles) and LD-Aag2 (squares) over a 7-day period. Assays were performed in triplicate. Unpaired Student's t tests were performed between groups to assess statistical significance. Standard deviations are shown.

Figure 2: LD-Aag2 cells are smaller but have equivalent total intracellular lipid concentration and reduced lipolysis.

(**A)** Representative images of Nile Red-stained C-Aag2, LD-Aag2, and LD-6X-Aag2 cells taken at 20X magnification. (**B)** Relative cell area of C-Aag2, LD-Aag2, and LD-6X-Aag2 cells measured using Nile Red staining and ImageJ software analysis. (**C)** Relative total intracellular lipid per cell of C-Aag2, LD-Aag2, and LD-6X-Aag2 determined by Nile Red pixel intensity and normalized per cell using Hoescht nuclear staining. ImageJ software was used for analysis. Ten unbiased digital photos were used for each condition. **(D)** Glycerol analysis of cell free

supernatants in C-Aag2 cells treated with C (squares) and LD (circles) cell culture media on days 1, 3, 5, and 7 post-treatment. Unpaired Student's t tests were performed between groups to assess statistical significance. Standard deviations are shown.

Figure 3: LD-Aag2 cells have equivalent ATP concentration.

An ATP assay was performed on C-Aag2 and LD-Aag2 cell lysates in triplicate three days post-feeding with their respective cell culture media. An unpaired Student's t test was performed between groups to assess statistical significance. Standard deviations are shown.

Figure 4: RNA-Seq analysis of C and LD-Aag2 cells reveals differentially expressed genes.

Volcano plot of upregulated and downregulated genes comparing uninfected C and LD-Aag2 cells.

Figure 5: g:Cost functional profiling reveals genes categories that are upregulated in LD-Aag2 cells.

The g:Cost Functional Profiling tool at g:Profiler was used to quantify related genes that were upregulated in LD-Aag2 cells. The number of genes that intersect with significant gene ontology (GO) categories related to **(A)** molecular function, **(B)** biological process, and **(C)** cellular compartment are shown.

Figure 6: g:Cost functional profiling reveals genes categories that are downregulated in LD-Aag2 cells.

The g:Cost Functional Profiling tool at g:Profiler was used to quantify related genes that were downregulated in LD-Aag2 cells. The number of genes that intersect with significant gene ontology (GO) categories related to **(A)** molecular function, **(B)** biological process, and **(C)** cellular compartment are shown.

Figure 7: DAVID Annotation Clustering reveals genes with catalytic activity and membrane components that are differentially expressed in LD-Aag2 cells.

(A) Upregulated LD-Aag2 genes associated with catalytic activity were grouped into 19 clusters (**B)** Upregulated LD-Aag2 genes associated with membrane or intrinsic component of membrane were grouped into 19 clusters.

Figure 8: Gene expression validation using uninfected and DENV-infected C and LD-Aag2 cells reveals a blunted innate immune response in LD-Aag2 cells.

(A-D) Representative genes were selected that had differential gene expression in the RNA-Seq dataset (i.e., AAEL007619, AAEL004863, AAEL005665, and AAEL014078). C and LD-Aag2 cells were infected with DENV and gene expression was determined 0, 12, and 24 hours post infection. Assays were performed in triplicate. Unpaired Student's t tests were performed between groups to assess statistical significance. Standard deviations are shown.

Figure 9: Infectivity assays reveal equivalent shedding of DENV in LD-Aag2 cells.

(A) viral RNA (vRNA) assay of cell-free supernatants taken from DENV-infected C-Aag2 cells 0, 1, 3, and 5 dpi **(B)** vRNA assay of cell-free supernatants taken from uninfected (M), and DENV-infected C-Aag2, LD-Aag2, and LD-6X-Aag2 cells 3 dpi. **(C)** Representative image of a DENV-positive focus. **(D)** Focus Forming Unit (FFU) assay showing production of DENV from C-Aag2, LD-Aag2, and LD-6X-Aag2 cells 3 days post-infection (dpi). FFU's were normalized per cells due to the slower growth rate of LD and LD-6X cells. Assays were performed in triplicate. Unpaired Student's t tests were performed between groups to assess statistical significance. Standard deviations are shown.

Supplemental Table 1

Click here to access/download
Supporting Information
Supplemental Table 1 LDvsC_DEG.csv

Supplemental Table 2

Click here to access/download
Supporting Information
Supplemental Table 2 LDvsC_DEG_Up.csv

Supplemental Table 3

Click here to access/download
Supporting Information
Supplemental Table 3 LDvsC_DEG_Down.csv

Supplemental Table 4

Click here to access/download

Supporting Information

Supplemental Table 4 gProfiler_Up_LD.csv

Supplemental Table 5

Click here to access/download

Supporting Information

Supplemental Table 5 gProfiler_Down_LD.csv

Click here to access/download
Supporting Information
Supplemental Table 6 primer sequences for RT-qPCR.docx

Response to Reviewers-

Reviewer comments

Two reviewers have noted that there is no replication of the RNAseq analysis. This has been discussed by the editors who view this as a substantive concern. Replication is important as the estimation of P values depends on the between-replicate variance, and replication is good scientific practice in any experiment. As these P values underpin a substantial component of this manuscript, a requirement for resubmission is that the RNAseq analysis is replicated.

Thank you. We agree that at least three biological replicates are important to add significance to the RNA-Seq dataset. We have included these data in the revised manuscript.

In addition, please include the SRA NCBI submission numbers for the raw RNA-Seq data.

The RNA-Seq .bam files have been submitted to NIH SRA. The submission number has been included in the manuscript - SUB12087207.

The title should be changed to make it clear this is work in cell culture and not insects.

We have changed the title so that it states "*Aedes aegypti* cells".

Reviewer #1:

Overall the methodologies utilized in this study are appropriate for the questions being addressed. I have one issue with the methods which is the use of a single sample for each condition in their RNA-seq analysis. The authors mention that the samples include 3 pooled in vitro replicates and I know that DESEQ2 can be used to estimate variance within a single sample by looking a read distributions over multiple loci, however a clearer explanation of this technique and supporting references are needed. The qPCR-based validation of the RNA-seq results confirm the findings, but it would be better to have this clarified in the materials and methods.

Thank you for your response. After careful review of our strategy, we agree that at least three biological replicates are important to add significance to the RNA-Seq dataset. We have included these data in the revised manuscript.

I feel that more information on the cell line used in the paper (Aag2) should be provided. The cell line was derived from Professor Erol Fikrig's lab, but there is little to no information on how the cell line was generated and what tissue/life stage it was generated from. These are important considerations as the metabolic capacities and responses of different cell types in the mosquito could show significant differences. That is not to say the results are not of interest. However, the observed responses should be contextualized with any information available for this cell line.

Thank you for your response. We have added historical information about the cell line in the Introduction. Specifically, we added that "The Aag2 cell line was generated from whole homogenized embryos. Cells within the culture exhibit differing morphologies, and it has been suggested that the varying morphologies indicate the presence of multiple embryonic and differentiated cell types. Aag2

cells are immunocompetent and persistently infected with a number of insect-specific viruses, including cell fusing agent virus (CFAV) and Phasi Charoen-like virus (PCLV) [31-33].

Reviewer #2:

The authors need to clearly state the hypothesis to be tested before each of the respective methods section. A brief sentence introducing the hypothesis (objective) being tested can be written just before describing the method. For example the statement "To investigate differential gene expression between uninfected or infected C and LD-Aag2 cells, we performed next generation RNA sequencing (RNA-Seq) analysis on both the uninfected and DENV2-infected C and LD-Aag2 cells." can be added on line 432. All other aspects of the methods have been well addressed.

Thank you for your response. We have added brief hypotheses in the results section, which provide context for each experiment. Details for each experiment are listed in the methods section. For instance, we wrote, “We hypothesized that Aag2 adapt to lipid-depleted conditions by changing expression of metabolism genes, which would reduce mitotic rate to match the reduction in catabolic substrate and promote de novo lipid biosynthesis. We tested this hypothesis by performing next generation RNA sequencing (RNA-Seq) analysis on C and LD-Aag2 cells and investigated the statistically significant differentially expressed genes (DEGs)” in the results. Specific details of RNA-Seq are in the methods.

Reviewer #3:

1. The statistics and such need better description. Is the data normal? What specific methods were used?
2. Single replicate RNA-seq can only be used in a limited setting. Did the qPCR values validate the RNA-seq based on a correlation analysis?

Thank you for your response. After careful review of our strategy, we agree that at least three biological replicates are important to add significance to the RNA-Seq dataset. We have included these data in the revised manuscript. We have not performed a correlation analysis for validation, although the additional biological replicates and follow-up RT-qPCR validation provide significant support that.

3. A GO clustering program (Revigo, etc.) needs to be used for interpretation.

GO molecular function, cellular component, and biological processes are shown in Figure 5.

4. Please display the data point on the bar charts for transparency or make data available.

Thank you for your response. We respectfully ask for more direction so that we can add the necessary data to bar charts. Is there a specific data point that you are interested in, and how would you like that represented?

5. Please include the NCBI submission numbers for the RNA-seq sets.

The RNA-Seq .bam files have been submitted to NIH SRA. The submission number has been included in the manuscript - SUB12087207.

Reviewer #1: The analyses and results match the analysis plan and are clearly presented. However, I have some issues with the presentation of the data shown in figure 5, 6, and 7. I feel these could be presented in an alternative graphical format that would significantly improve their readability.

Thank you for your response. These data have also been provided as horizontal bar graphs as requested and as Supplemental Tables, which will allow readers access to a second method to read and analyze the data. The text size is larger. We will work with copyeditors to ensure that the figures and text size are at the appropriate size for readers.

Reviewer #2: Font size on figure 4,5,6 and 7 needs to be improved for clarity.
All other aspects of the results are well presented.

Thank you for your response. These data have also been provided as horizontal bar graphs as requested and as Supplemental Tables, which will allow readers access to a second method to read and analyze the data. The text size is larger. We will work with copyeditors to ensure that the figures and text size are at the appropriate size for readers.

Reviewer #3: 1. Text in many figures will be too small. Please revise.

Thank you for your response. These data have also been provided as horizontal bar graphs as requested and as Supplemental Tables, which will allow readers access to a second method to read and analyze the data. The text size is larger. We will work with copyeditors to ensure that the figures and text size are at the appropriate size for readers.

Reviewer #1: I feel that the conclusions of the manuscript are appropriate and supported by the data presented. However, I think that the limitations of using a cell line to extrapolate to what is happening in vivo need to be considered in the discussion. This weakness is not discussed in depth and the limitations should be addressed to temper the conclusions.

Thank you for your response. We have changed the title to include "*Aedes aegypti* cells". We also state in the discussion that, "we developed an *in vitro* model system that consisted of feeding Aag2 cells with complete (C) and lipid-depleted (LD) cell culture media. In this context, C-Aag2 cells represent a blood fed mosquito that is replete with factors derived from vertebrate serum."

Reviewer #2: The discussion is well supported by the data presented and has enhanced our understanding of targeting lipid metabolism as a method of controlling mosquitos.

Thank you.

Reviewer #3: The RNAseq being only single replicate and timepoint is a concern and this needs to be discussed.

Thank you. We agree that at least three biological replicates are important to add significance to the RNA-Seq dataset. We have included these data in the revised manuscript.

Reviewer #1: Figures 5, 6, and 7 are presented as pie charts, which are not very informative. Pie charts are not optimal for use in interpretation of differences in categories due to difficulties in visual

perception of area. These figures would be much easier to interpret as horizonal bar charts. This would provide more room for larger labels and easier comparison of differences between categories.

Thank you for your response. These data have also been provided as horizontal bar graphs as requested and as Supplemental Tables, which will allow readers access to a second method to read and analyze the data. The text size is larger. We will work with copyeditors to ensure that the figures and text size are at the appropriate size for readers.

Reviewer #2: As indicated on the Annotated MS

Reviewer #3: NA

Reviewer #1:

However, as mentioned above, the authors need to limit how far these results can be interpreted as they are derived from a single cell line rather than a whole organism.

Thank you for your response. We have changed the title to include "*Aedes aegypti* cells". We also state in the discussion that, "we developed an *in vitro* model system that consisted of feeding Aag2 cells with complete (C) and lipid-depleted (LD) cell culture media. In this context, C-Aag2 cells represent a blood fed mosquito that is replete with factors derived from vertebrate serum."

The results in terms of cellular response to lipid deficiency are interesting, but I think a section of the discussion should be added to address the limitations of the system and inclusion of more background on the biology/derivation of the cell line that is being used.

Thank you for your response. We have added historical information about the cell line in the Introduction. Specifically, we added that "The Aag2 cell line was generated from whole homogenized embryos. Cells within the culture exhibit differing morphologies, and it has been suggested that the varying morphologies indicate the presence of multiple embryonic and differentiated cell types. Aag2 cells are immunocompetent and persistently infected with a number of insect-specific viruses, including cell fusing agent virus (CFAV) and Phasi Charoen-like virus (PCLV) [31-33].

I would also like to see a clearer explanation of how the statistical analysis of the RNA-seq data was performed using only a single pooled sample per condition with inclusion of published validation of this methodology.

Thank you. After careful review of our strategy, we agree that at least three biological replicates are important to add significance to the RNA-Seq dataset. We have included these data in the revised manuscript.

Finally, I would recommend that the output of the gene ontology analyses be converted from a pie chart format to a horizontal bar or line plot to make interpretation easier for the reader.

Thank you for your response. These data have also been provided as horizontal bar graphs as requested and as Supplemental Tables, which will allow readers access to a second method to read and analyze the data. The text size is larger. We will work with copyeditors to ensure that the figures and text size are at the appropriate size for readers.

I could not find a SRA or Bioproject number associated with the RNA-seq data for public accessibility. This needs to be included.

The RNA-Seq .bam files have been submitted to NIH SRA. The submission number has been included in the manuscript - SUB12087207.

Reviewer #2: The authors have articulated what is currently known on the effect of vertebrate and invertebrate lipids on mosquito infectivity of viruses, particularly DENV. The objectives of this study have been designed carefully to address the question of whether blood feeding introduces vertebrate-specific factors into the mosquito that may be important for regulating interactions between the mosquito vector and DENV. Further, the authors have introduced an innovative technique where a vertebrate lipid-depleted Ae. aegypti cell line was used to investigate the effect of chronic depletion of vertebrate lipids normally present in a blood meal and insect cell culture medium on cell growth and virus infection. Relevant comparisons have been done to previous studies which further reinforce the findings. The authors have explicitly stated their main findings which provide opportunities for future studies in targeting lipid metabolism as control methods against mosquito populations and the pathogens the transmit.

Thank you for your response.

Reviewer #3: The study is interesting but will require substantial revisions.

Thank you for your response. We have repeated the RNA-Seq analysis with biological triplicates, added information about the Aag2 cell line, and modified figures for clarity.

Click here to access/download
Revised Article with Changes Highlighted
Compare and Contrast.docx
