# Supplementary material for: Chronic depletion of vertebrate lipids in Aedes aegypti cells dysregulates lipid metabolism and inhibits innate immunity without altering dengue infectivity
Source: PLoS Negl Trop Dis. 2022 Oct 24;16(10):e0010890. doi: 10.1371/journal.pntd.0010890 (PMC9632908; doi:10.1371/journal.pntd.0010890)
Supplement: Attachment — Submitted filename: Response to Reviewers.docx [file pntd.0010890.s008.docx]

Response to Reviewers-

Reviewer comments

Two reviewers have noted that there is no replication of the RNAseq analysis. This has been discussed by the editors who view this as a substantive concern. Replication is important as the estimation of P values depends on the between-replicate variance, and replication is good scientific practice in any experiment. As these P values underpin a substantial component of this manuscript, a requirement for resubmission is that the RNAseq analysis is replicated.

Thank you. We agree that at least three biological replicates are important to add significance to the RNA-Seq dataset. We have included these data in the revised manuscript.

In addition, please include the SRA NCBI submission numbers for the raw RNA-Seq data.

The RNA-Seq .bam files have been submitted to NIH SRA. The submission number has been included in the manuscript - SUB12087207.

The title should be changed to make it clear this is work in cell culture and not insects.

We have changed the title so that it states “*Aedes aegypti* cells”.

Reviewer #1:

Overall the methodologies utilized in this study are appropriate for the questions being addressed. I have one issue with the methods which is the use of a single sample for each condition in their RNA-seq analysis. The authors mention that the samples include 3 pooled in vitro replicates and I know that DESEQ2 can be used to estimate variance within a single sample by looking a read distributions over multiple loci, however a clearer explanation of this technique and supporting references are needed. The qPCR-based validation of the RNA-seq results confirm the findings, but it would be better to have this clarified in the materials and methods.

Thank you for your response. After careful review of our strategy, we agree that at least three biological replicates are important to add significance to the RNA-Seq dataset. We have included these data in the revised manuscript.

I feel that more information on the cell line used in the paper (Aag2) should be provided. The cell line was derived from Professor Erol Fikrig's lab, but there is little to no information on how the cell line was generated and what tissue/life stage it was generated from. These are important considerations as the metabolic capacities and responses of different cell types in the mosquito could show significant differences. That is not to say the results are not of interest. However, the observed responses should be contextualized with any information available for this cell line.

Thank you for your response. We have added historical information about the cell line in the Introduction. Specifically, we added that “The Aag2 cell line was generated from whole homogenized embryos. Cells within the culture exhibit differing morphologies, and it has been suggested that the varying morphologies indicate the presence of multiple embryonic and differentiated cell types. Aag2 cells are immunocompetent and persistently infected with a number of insect-specific viruses, including cell fusing agent virus (CFAV) and Phasi Charoen-like virus (PCLV) [31-33].

Reviewer #2:

The authors need to clearly state the hypothesis to be tested before each of the respective methods section. A brief sentence introducing the hypothesis (objective) being tested can be written just before describing the method. For example the statement "To investigate differential gene expression between uninfected or infected C and LD-Aag2 cells, we performed next generation RNA sequencing (RNA-Seq) analysis on both the uninfected and DENV2-infected C and LD-Aag2 cells." can be added on line 432. All other aspects of the methods have been well addressed.

Thank you for your response. We have added brief hypotheses in the results section, which provide context for each experiment. Details for each experiment are listed in the methods section. For instance, we wrote, “We hypothesized that Aag2 adapt to lipid-depleted conditions by changing expression of metabolism genes, which would reduce mitotic rate to match the reduction in catabolic substrate and promote de novo lipid biosynthesis. We tested this hypothesis by performing next generation RNA sequencing (RNA-Seq) analysis on C and LD-Aag2 cells and investigated the statistically significant differentially expressed genes (DEGs)” in the results. Specific details of RNA-Seq are in the methods.

Reviewer #3:

1. The statistics and such need better description. Is the data normal? What specific methods were used?

2. Single replicate RNA-seq can only be used in a limited setting. Did the qPCR values validate the RNA-seq based on a correlation analysis?

Thank you for your response. After careful review of our strategy, we agree that at least three biological replicates are important to add significance to the RNA-Seq dataset. We have included these data in the revised manuscript. We have not performed a correlation analysis for validation, although the additional biological replicates and follow-up RT-qPCR validation provide significant support that.

3. A GO clustering program (Revigo, etc.) needs to be used for interpretation.

GO molecular function, cellular component, and biological processes are shown in Figure 5.

4. Please display the data point on the bar charts for transparency or make data available.

Thank you for your response. We respectfully ask for more direction so that we can add the necessary data to bar charts. Is there a specific data point that you are interested in, and how would you like that represented?

5. Please include the NCBI submission numbers for the RNA-seq sets.

The RNA-Seq .bam files have been submitted to NIH SRA. The submission number has been included in the manuscript - SUB12087207.

Reviewer #1: The analyses and results match the analysis plan and are clearly presented. However, I have some issues with the presentation of the data shown in figure 5, 6, and 7. I feel these could be presented in an alternative graphical format that would significantly improve their readability.

Thank you for your response. These data have also been provided as horizontal bar graphs as requested and as Supplemental Tables, which will allow readers access to a second method to read and analyze the data. The text size is larger. We will work with copyeditors to ensure that the figures and text size are at the appropriate size for readers.

Reviewer #2: Font size on figure 4,5,6 and 7 needs to be improved for clarity.

All other aspects of the results are well presented.

Thank you for your response. These data have also been provided as horizontal bar graphs as requested and as Supplemental Tables, which will allow readers access to a second method to read and analyze the data. The text size is larger. We will work with copyeditors to ensure that the figures and text size are at the appropriate size for readers.

Reviewer #3: 1. Text in many figures will be too small. Please revise.

Thank you for your response. These data have also been provided as horizontal bar graphs as requested and as Supplemental Tables, which will allow readers access to a second method to read and analyze the data. The text size is larger. We will work with copyeditors to ensure that the figures and text size are at the appropriate size for readers.

Reviewer #1: I feel that the conclusions of the manuscript are appropriate and supported by the data presented. However, I think that the limitations of using a cell line to extrapolate to what is happening in vivo need to be considered in the discussion. This weakness is not discussed in depth and the limitations should be addressed to temper the conclusions.

Thank you for your response. We have changed the title to include “*Aedes aegypti* cells”. We also state in the discussion that, “we developed an *in vitro* model system that consisted of feeding Aag2 cells with complete (C) and lipid-depleted (LD) cell culture media. In this context, C-Aag2 cells represent a blood fed mosquito that is replete with factors derived from vertebrate serum.”

Reviewer #2: The discussion is well supported by the data presented and has enhanced our understanding of targeting lipid metabolism as a method of controlling mosquitos.

Thank you.

Reviewer #3: The RNAseq being only single replicate and timepoint is a concern and this needs to be discussed.

Thank you. We agree that at least three biological replicates are important to add significance to the RNA-Seq dataset. We have included these data in the revised manuscript.

Reviewer #1: Figures 5, 6, and 7 are presented as pie charts, which are not very informative. Pie charts are not optimal for use in interpretation of differences in categories due to difficulties in visual perception of area. These figures would be much easier to interpret as horizonal bar charts. This would provide more room for larger labels and easier comparison of differences between categories.

Thank you for your response. These data have also been provided as horizontal bar graphs as requested and as Supplemental Tables, which will allow readers access to a second method to read and analyze the data. The text size is larger. We will work with copyeditors to ensure that the figures and text size are at the appropriate size for readers.

Reviewer #2: As indicated on the Annotated MS

Reviewer #3: NA

Reviewer #1:

However, as mentioned above, the authors need to limit how far these results can be interpreted as they are derived from a single cell line rather than a whole organism.

Thank you for your response. We have changed the title to include “*Aedes aegypti* cells”. We also state in the discussion that, “we developed an *in vitro* model system that consisted of feeding Aag2 cells with complete (C) and lipid-depleted (LD) cell culture media. In this context, C-Aag2 cells represent a blood fed mosquito that is replete with factors derived from vertebrate serum.”

The results in terms of cellular response to lipid deficiency are interesting, but I think a section of the discussion should be added to address the limitations of the system and inclusion of more background on the biology/derivation of the cell line that is being used.

Thank you for your response. We have added historical information about the cell line in the Introduction. Specifically, we added that “The Aag2 cell line was generated from whole homogenized embryos. Cells within the culture exhibit differing morphologies, and it has been suggested that the varying morphologies indicate the presence of multiple embryonic and differentiated cell types. Aag2 cells are immunocompetent and persistently infected with a number of insect-specific viruses, including cell fusing agent virus (CFAV) and Phasi Charoen-like virus (PCLV) [31-33].

I would also like to see a clearer explanation of how the statistical analysis of the RNA-seq data was performed using only a single pooled sample per condition with inclusion of published validation of this methodology.

Thank you. After careful review of our strategy, we agree that at least three biological replicates are important to add significance to the RNA-Seq dataset. We have included these data in the revised manuscript.

Finally, I would recommend that the output of the gene ontology analyses be converted from a pie chart format to a horizontal bar or line plot to make interpretation easier for the reader.

Thank you for your response. These data have also been provided as horizontal bar graphs as requested and as Supplemental Tables, which will allow readers access to a second method to read and analyze the data. The text size is larger. We will work with copyeditors to ensure that the figures and text size are at the appropriate size for readers.

I could not find a SRA or Bioproject number associated with the RNA-seq data for public accessibility. This needs to be included.

The RNA-Seq .bam files have been submitted to NIH SRA. The submission number has been included in the manuscript - SUB12087207.

Reviewer #2: The authors have articulated what is currently known on the effect of vertebrate and invertebrate lipids on mosquito infectivity of viruses, particularly DENV. The objectives of this study have been designed carefully to address the question of whether blood feeding introduces vertebrate-specific factors into the mosquito that may be important for regulating interactions between the mosquito vector and DENV. Further, the authors have introduced an innovative technique where a vertebrate lipid-depleted Ae. aegypti cell line was used to investigate the effect of chronic depletion of vertebrate lipids normally present in a blood meal and insect cell culture medium on cell growth and virus infection. Relevant comparisons have been done to previous studies which further reinforce the findings. The authors have explicitly stated their main findings which provide opportunities for future studies in targeting lipid metabolism as control methods against mosquito populations and the pathogens the transmit.

Thank you for your response.

Reviewer #3: The study is interesting but will require substantial revisions.

Thank you for your response. We have repeated the RNA-Seq analysis with biological triplicates, added information about the Aag2 cell line, and modified figures for clarity.
